# miR-30e* is overexpressed in prostate cancer and promotes NF-κB-mediated proliferation and tumor growth

**DOI:** 10.18632/oncotarget.18795

**Published:** 2017-06-28

**Authors:** Shawn M. Egan, Ellen Karasik, Leigh Ellis, Sandra O. Gollnick

**Affiliations:** ^1^ Department of Immunology, Roswell Park Cancer Institute, Buffalo, New York 14263, USA; ^2^ Department of Pharmacology and Therapeutics, Roswell Park Cancer Institute, Buffalo, New York 14263, USA; ^3^ Department of Oncologic Pathology, Dana-Farber Cancer Institute, Boston, MA 02215, USA; ^4^ Department of Cell Stress Biology, Roswell Park Cancer Institute, Buffalo, New York 14263, USA

**Keywords:** NF-κB, microRNA, prostate cancer, cyclin D1

## Abstract

According to the CDC prostate cancer (CaP) has the highest incidence and second highest mortality rate amongst cancers in American men. Constitutive NF-κB activation is a hallmark of CaP and this pathway drives many pro-tumorigenic characteristics of CaP cells, including cell proliferation and survival. An activated NF-κB gene signature is predictive of CaP progression and biochemical recurrence following therapeutic intervention. However, the mechanisms that perpetuate NF-κB activation are incompletely understood. Genes that control NF-κB activity are rarely mutated in CaP suggesting that epigenetic mechanisms may contribute to constitutive NF-κB activation. microRNAs (miRs) epigenetically regulate many genes involved with NF-κB activation. IκBα is a direct inhibitor of NF-κB; it binds to and sequesters NF-κB in the cytoplasm resulting in functional inhibition. IκBα is a target gene of miR-30e* yet the expression and oncological impact of miR-30e* in CaP is unknown. We report that miR-30e* expression is elevated in multiple murine models of CaP and is most pronounced in late stage disease. miR-30e* drives CaP proliferation and tumor growth through inhibition of IκBα, which results in chronic activation of NF-κB. Additionally, we show that inhibition of miR-30e* improves chemotherapeutic control of CaP. Thus, miR-30e* may prove to be a novel clinical target whose inhibition leads to decreased CaP cell proliferation and sensitization of CaP cells to chemotherapeutics.

## INTRODUCTION

Prostate cancer (CaP) is the most commonly diagnosed male cancer worldwide. Recent data from the American Cancer Society predicts that the estimated deaths from CaP are higher than any other cancer, except lung [1]. CaP is managed with a combination of prostatectomy, radiation, chemotherapeutics and androgen ablation [2]. While treatment of CaP can be curative, patients may experience biochemical recurrence and develop hormone refractory disease [2]. There are currently no curative options for hormone refractory disease; thus, new and novel treatment options are critically needed.

The precise mechanisms that drive the development of hormone refractory disease remain unknown; however, chronic inflammation and aberrant proliferation of prostate epithelial cells are believed to play a substantial role [3–5]. Nuclear factor-κB (NF-κB) activates cellular programs critical for cell survival, proliferation, and the induction of a robust inflammatory response through the expression of a number of genes [6, 7]. NF-κB is a family of highly conserved homo and heterodimer transcription factors that are ubiquitously expressed in various cell types [8, 9]. The NF-κB family consists of 5 proteins; p65 (Rel A), c-Rel, Rel B, p50 (NFKB1) and p52 (NFKB2) [9]. Three separate pathways can activate NF-κB: the canonical pathway, the non-canonical pathway and the atypical pathway [6, 7]. The canonical pathway involves the activation of p65:p50 heterodimers by IKKβ. In the non-canonical pathway p52:Rel B heterodimers are activated when IKKα homodimers process p100 into active p52. The atypical pathway is characterized by IKK-independent dissociation of NF-κB from nuclear factor of κ light polypeptide gene enhancer in B-cells inhibitor, alpha (IκBα).

Numerous factors regulate the initiation and duration of canonical NF-κB activation; the most dominant being the sequestration of NF-κB in the cytoplasm by IκBα [7, 8, 10]. In resting cells, IκBα restrains NF-κB in the cytoplasm; following an activation cue, IκBα becomes phosphorylated and subsequently ubiquitinated leading to its degradation [7, 8, 10]. Without the restraint of IκBα, NF-κB is able to translocate to the nucleus and promote target gene transcription. IκBα itself is an NF-κB target gene [9]. Thus, NF-κB activation drives a negative feedback loop resulting in NF-κB being re-sequestered in the cytoplasm [9, 8, 11]. In spite of this negative feedback pathway, NF-κB is constitutively activated in many diseases including CaP [6, 8–11]. Constitutive activation of the canonical NF- κB pathway is associated with a worse prognosis [12–14] and has been documented to be critical for chemotherapeutic resistance in CaP [15]. Furthermore, the expression of the NF-κB target genes, IL-6, VEGF, MMP9, and TNF-α are positively correlated with the extent and severity of CaP. These genes also contribute to CaP initiation, growth, progression, and metastasis through the induction of chronic inflammation and angiogenesis [4, 16, 17]. Cyclin D1, another NF-κB target gene, is critical for continued CaP cell proliferation [16, 18]. Although the role that NF-κB plays in CaP is well appreciated, the precise mechanisms facilitating constitutive NF-κB activation remain unknown. The mutation rate of genes encoding NF-κB and the molecules regulating its activation, such as IκBα, is low in clinical CaP samples [19, 20]. Thus, epigenetics may contribute to constitutive NF-κB activation.

microRNAs (miRs) are small RNA molecules approximately 20-25 nucleotides in length [21]. miRs bind to complementary target regions located predominately in the 3′ untranslated regions of mRNA transcripts [21]. miRs regulate the expression of their target mRNA by translational repression and/or facilitating mRNA decay [22]. In CaP, global miR dysregulation correlates with disease development, invasion and metastasis [23, 24]. Mounting evidence suggests that aberrant expression of miRs contributes to cell hyper-proliferation, apoptosis, chronic inflammation, and the induction of the stress response [23–25].

NF-κB activation is both directly and indirectly regulated by miRs. miRs specifically target NF-κB mRNA transcripts as well as alter the expression of genes that coordinate its activation [9, 26, 27, 28]. An example is miR-30e* which inhibits IκBα [27]. In human glioma hyper-expression of miR-30e* constitutively drives NF-κB activation [27]. Yet the expression and biological impact of miR-30e* in CaP is unknown.

In this study we demonstrate that miR-30e* is over-expressed in murine models of CaP. We show that miR-30e* drives CaP cell proliferation by targeting IκBα. Mechanistically, miR-30e* increases NF-κB activation, increases the expression of cyclin D1 which prompts the phosphorylation of Rb, a critical regulator of CaP proliferation. Evaluation of the clinical significance of this finding revealed that targeting the miR30e*: IκBα axis can control prostate tumor growth. Additionally, pretreating CaP cells with a miR-30e* inhibitor sensitizes the cells to the chemotherapeutic docetaxel.

## RESULTS

### miR-30e* is overexpressed in murine models of prostate cancer

**Figure 1:**
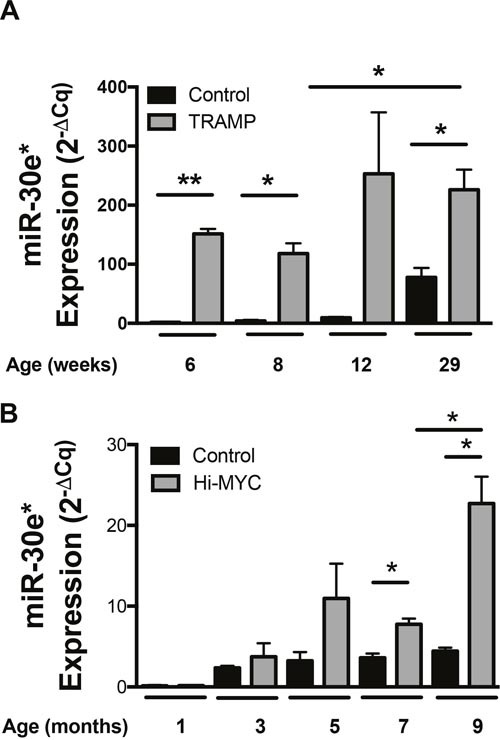
miR-30e* expression is elevated in CaP **(A)** Whole prostates were harvested from TRAMP mice at 6-, 8-, 12 and 29-weeks of age and corresponding age matched control C57BL/6J mice (n = 3). **(B)** Prostates were also harvested from Hi-MYC mice along with wild type FVB age matched control mice (n = 2). Prostates were analyzed for miR-30e* and U6 snRNA expression via qRT-PCR. Raw data was analyzed and displayed in graph using the 2^−dCq^ formula. Welch's t-test (A) and Student t-tests were performed (B), Error bars represent SEM; * *P* ≤ 0.05, ** *P* ≤ 0.01.

The expression of miR-30e* was examined in two autochthonous experimental mouse models of CaP; the TRansgenic Adenocarcinoma of the Mouse Prostate (TRAMP) model system and the HI-Myc model. The TRAMP model system is a probasin driven SV-40 TAg transformation model that manifests as prostate epithelial specific transformation [29]. The probasin promoter is activated in response to androgen, which is first produced when mice reach puberty at 4 weeks of age. Mice consistently develop prostatic intraepithelial neoplasia (PIN) by 6 weeks of age and then progress to macroscopic disease by 12-29 weeks of age [29]. miR-30e* expression was significantly higher in TRAMP prostates when compared to syngeneic age-matched C57BL/6 control prostates at multiple age points (Figure 1A; *P ≤ 0.05). To validate that elevated miR-30e* expression in CaP was not a model specific phenomenon, miR-30e* expression in the Hi-MYC transgenic CaP model [30] was also analyzed (Figure 1B). Hi-MYC mice develop PIN as early as 2 weeks of age and progress to macroscopic cancer by 6 months [31]. miR-30e* expression was significantly elevated in prostates isolated from Hi-MYC transgenic mice relative to aged-matched control prostates isolated from FVB mice. At ages which have been shown to be tumor bearing miR-30e* expression was significantly elevated compared to control mice (7 & 9 months; *P ≤ 0.05). There was also a significant difference between 7 and 9 months in experimental mice echoing the TRAMP data suggesting miR-30e* may increase with disease progression (Figure 1B; 7 vs 9 months, *P ≤ 0.05).

### miR-30e* regulates prostate cancer cell viability

**Figure 2:**
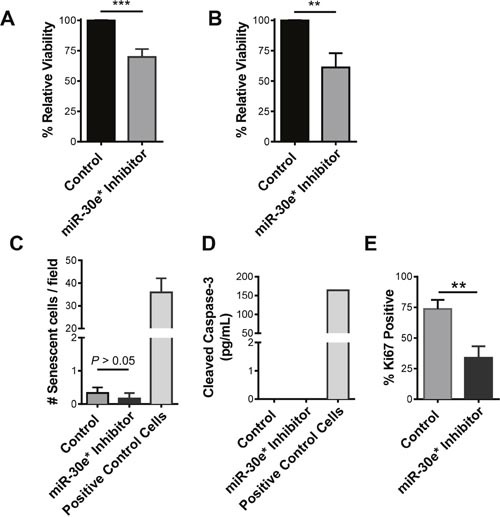
miR-30e* regulates CaP cell proliferation **(A)** C2H cells or **(B)** PC3M cells were transfected with either miR-30e* inhibitor oligos (■) or control scramble oligos. Twenty-four and forty-eight hours later MTT assays were performed. Results are reported as % viability relative to viability observed in cells transfected with control scramble oligos; each time point of the experiments was repeated a minimum of 4 times. Welch's t-tests were performed, Error bars represent SEM;* *P* ≤ 0.05, ** *P* ≤0.01, *** *P* ≤ 0.001, **** *P* ≤0.0001. **(C)** Cell senescence was tested by staining either control or miR-30e* inhibited C2H cells for β-galactosidase, hydrogen peroxide treated fibroblasts were used as a positive control (Positive Control). Positively stained cells were analyzed in three separate 200x fields of view; counts were repeated 3 times and the average of the counts was recorded. Results are reported as # of senescent cells / field. Welch's t-tests were performed, error bars represent SEM; n = 3, *P >* 0.05. **(D)** Cell apoptosis was tested by detecting cleaved caspase-3 via ELISA in control or miR-30e* inhibited C2H cells, TSA treated JAR cells were used as a positive control (Positive Control Cells). Results are reported as pg/mL of cleaved caspase 3. Welch's t-tests were performed, error bars represent SEM; n = 3, *P >* 0.05. **(E)** Cell proliferation was tested by IHC staining of Ki67 in control or miR-30e* inhibited C2H cells. Positively stained cells were analyzed using a light microscope in three separate 200x fields of view; counts were repeated 3 times and the average of the counts is presented as % of Ki67 positive cells. Welch's t-tests were performed, error bars represent SEM; n = 3, * *P ≤ 0.05*.

Inhibition of miR-30e* reduced the viability of TRAMP C2H tumor cells, a cell line derived from the TRAMP model (Figure 2A; ****P ≤ 0.001). Similar results were observed when miR-30e* was inhibited in the human CaP cell line PC3M (Figure 2B; day 1: **P ≤ 0.01 and day 2: *P ≤ 0.05). Confirmation of miR-30e* inhibition was performed in both TRAMP C2H and PC3M cells (Supplementary Figure 1A & 1B; *P ≤ 0.05 ***P ≤ 0.001). To determine how miR-30e* regulated CaP cell viability, the effects of miR-30e* inhibition on cell senescence, death and proliferation were tested. Inhibition of miR-30e* had no effect on the expression of senescence-associated β-galactosidase (Figure 2C; *P > 0.05) or cleaved caspase-3 (Figure 2D; *P > 0.05) suggesting that miR-30e* is not altering cell viability by inhibiting the percentage of cells that enter senescence or altering the rate of apoptotic cell death. miR-30e* inhibition did however significantly reduce the percentage Ki67 expressing cells (Figure 2E; **P ≤ 0.01) suggesting that the decrease in the cell viability following miR-30e* inhibition (Figure 2A & 2B) was due in part to a reduction in proliferation.

### miR-30e* regulates NF-κB activity which is essential for prostate cancer cell viability

**Figure 3:**
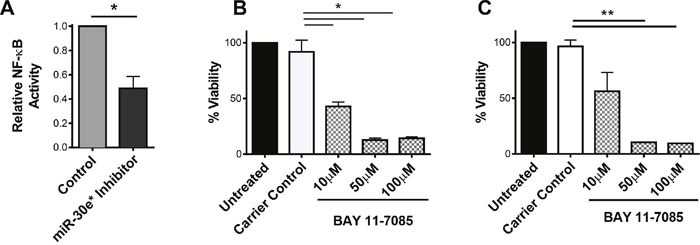
miR-30e* regulates NF-κB activity which is essential for prostate cancer cell viability **(A)** To evaluate NF-κB activation, an NF-κB luciferase reporter construct was transfected into C2H cells. Luciferase was then evaluated in control and miR-30e* inhibited cells 24 hours post inhibition. Results are depicted as NF-κB activity relative to control experimental group. Welch's t-tests were performed, error bars represent SEM; n = 4, * *P ≤ 0.05*. To assess whether NF-κB regulates prostate cancer viability TRAMP C2H **(B)** and PC3M **(C)** cell viability was assessed following a 24 hour treatment with 10 μM, 50 μM and 100 μM Bay 11-7085 treatment via MTT analysis. Equal volume ethanol carrier was used as a control. Welch's t-tests were performed, error bars represent SEM, n = 3, *P ≤ 0.05 and **P ≤ 0.01.

IκBα is a dominant inhibitor of the canonical NF-κB activation pathway. IκBα sequesters p65:p50 in the cytoplasm and is a confirmed target of miR-30e* [27]. Inhibition of miR-30e* led to a significant decrease in NF-κB activity (Figure 3A; *P ≤ 0.05). To assess whether NF-κB activation regulates prostate cancer cell viability and proliferation, TRAMP C2H and PC3M cells were treated separately with the NF-κB inhibitor; Bay 11-7085 which prevents the phosphorylation and subsequent degradation of IκBα (Figure 3B & 3C). Treatment with Bay 11-7085 significantly reduced the percentage of viable cells in both TRAMP C2H and PC3M cells (Figure 3B; ** P ≤ 0.01, Figure 3C; *P ≤ 0.05). Confirmation that Bay 11-8075 effectively inhibited IκBα phosphorylation following LPS stimulation in both TRAMP C2H and PC3M cells is depicted in Supplementary Figure 2.

### miR-30e* regulates prostate cancer cell proliferation and tumor growth through IκBα

**Figure 4:**
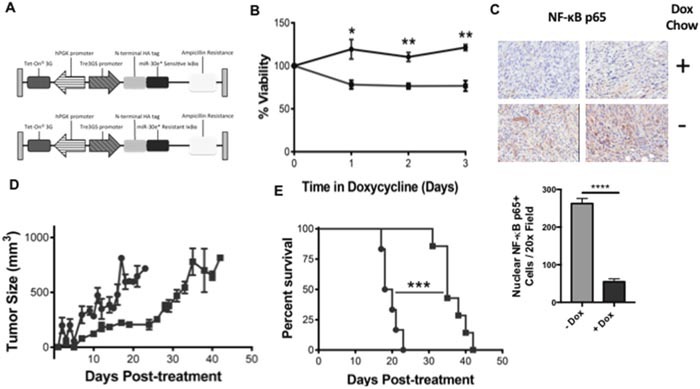
miR-30e* regulates prostate cancer cell proliferation and tumor growth through IκBα **(A)** Plasmid maps for doxycycline inducible N-terminal HA tagged miR-30e* sensitive IκBα and miR-30e* resistant IκBα pTetOne plasmids. **(B)** To evaluate whether the miR-30e*: IκBα interaction regulates CaP cell proliferation, C2H cells were transfected with a doxycycline inducible vector that expresses either miR-30e* sensitive or resistant IκBα. Cell viability was assessed in both miR-30e* sensitive (●) and resistant (■) clones following stimulation with 100 ng/mL doxycycline hyclate via MTT assay. Results are depicted as fold change relative cells not treated with doxycycline. Welch's t-tests were performed, error bars represent SEM; n ≥ 3,* *P* ≤ 0.05, ** *P* ≤0.01. To determine if the specific targeting of IκBα by miR-30e* regulated NF-κB p65 activation miR30e* resistant IκBα C2H cells were administered subcutaneously in C57BL/6 mice **(C)**. Once the tumors reached 100mm3, mice were randomized and initiated on either dox chow or remained on control chow. Tumor were explanted once the tumor volume reached 800mm3 and nuclear NF-κB p65 was quantified via IHC. Five sections were quantified from each tumor (n=4, *P* ≤ 0.001). Normal (●) or doxycycline containing (■) chow tumors were monitored and tumor growth was assessed. Results are depicted as tumor growth (mm^3^) after doxycycline treatment **(D)** and percent survival **(E)**. Welch's t-tests were performed, error bars represent SEM; n ≥ 6, *** *P* ≤ 0.001.

To determine whether the effects of miR-30e* inhibition on CaP cell proliferation were due to the direct inhibition of IκBα by miR-30e*, TRAMP C2H cells were transfected with a plasmid encoding a doxycycline inducible miR-30e* resistant IκBα-HA fusion protein (Figure 4A). Mutation of this miR-30e* seed sequence in the 3’UTR of IκBα was previously described by Jiang *et al* [27]. Functionality and doxycycline induction of the IκBα-HA fusion protein are shown in Supplementary Figure 3A-3C. miR-30e* resistant IκBα TRAMP C2H cells were treated *in vitro* with doxycycline and viability was monitored via MTT assay. TRAMP C2H cells transfected with wild type IκBα were used as a control. Cells expressing miR-30e* resistant IκBα exhibited a significant reduction in proliferation (Figure 4B; day 1: *P ≤ 0.05, day 2: *P ≤ 0.05 and day 3: **P ≤ 0.01). To test whether the targeting of IκBα by miR-30e* regulates NF-κB p65 nuclear translocation *in vivo* TRAMP C2H cells expressing miR-30e* resistant IκBα were injected subcutaneously into male C57BL/6 mice. When the tumors reached 100mm3 the mice were randomized and fed either doxycycline chow or remained on normal chow. Doxycycline chow induced miR-30e* resistant IκBα while mice on normal chow maintained normal IκBα in the CaP cells. Tumors were explanted and assessed for nuclear NF-κB p65 via IHC (Figure 4C). miR-30e* resistant IκBα C2H tumors displayed a significant reduction in nuclear NF-κB p65 relative to tumors from control chow fed mice (Figure 4C; **** *P* ≤ 0.001). This data confirms previous findings from Jiang et al. suggesting miR-30e* positively regulates NF- κB activation through IκBα. To determine whether prostate tumor growth was negatively affected by inhibiting the interaction of miR-30e* with IκBα, miR-30e* resistant IκBα tumor growth was assessed and compared to tumors from mice fed with normal control chow. TRAMP C2H tumor growth was delayed in mice that were fed doxycycline chow (Figure 4D) and the survival of these mice was significantly improved (Figure 4E; *P* ≤ 0.005). Doxycycline induction of miR-30e* resistant IκBα in the tumors was confirmed by western blot (Supplementary Figure 4).

### miR-30e* regulates cyclin D1 expression

**Figure 5:**
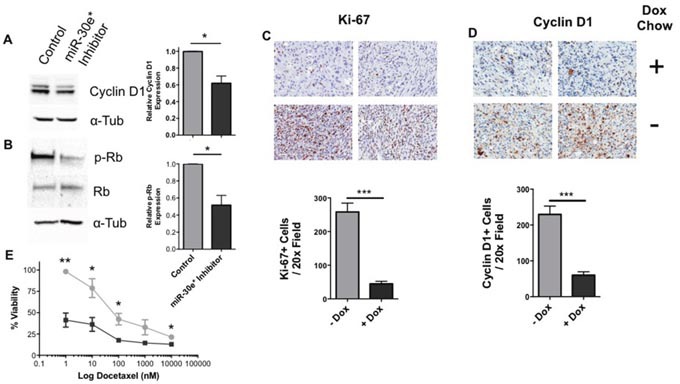
The miR-30e*: IκBα axis regulates cyclin D1 and proliferation *in vivo* The expression of cyclin D1 **(A)** and Rb (total and phosphorylated) **(B)** were evaluated in C2H cells treated with or without miR-30e* inhibitor for 24h via western blot analysis. Representative blots are shown on the right; summary results are depicted as expression relative to control C2H cells. Welch's t-tests were performed, error bars represent SEM; n ≥ 4, * *P ≤* 0.05. To assess if miR-30e* regulates prostate cancer cell proliferation and the expression of cyclin D1 *in vivo* IHC was performed. miR30e* resistant IκBα C2H cells were administered subcutaneously in C57BL/6 mice. When the tumors had established and reached 100mm^3^, normal (●) or doxycycline containing (■) chow was administered, tumors were harvested, formalin fixed and stained for Ki-67 **(C)** and cyclin D1 **(D)** when they reached 800 mm^3^. Images (C-D) represent IHC staining from 2x representative tumors from both normal and doxyxline chow fed mice and are displayed at 20x. **(E)** C2H cells were treated with either docetaxel alone (●) or in combination with miR-30e* inhibitor (■) and viability was assessed via MTT assays. Results are depicted as % viable (absorbance value of experimental relative to untreated TRAMP C2H cells). Welch's t-tests were performed, error bars represent SEM; n = 4, * *P* ≤ 0.05, ** *P* ≤ 0.01.

NF-κB affects cell proliferation through cyclin D1 [32], which regulates the G1-S phase transition [33]. Consistent with decreased NF-κB activity and cell proliferation, a significant reduction in cyclin D1 protein expression was observed *in vitro* in miR-30e* inhibited TRAMP C2H cells (Figure 5A; *P ≤ 0.05). Cyclin D1 in complex with cyclin-dependent kinase (CDK) 4/6 phosphorylate the tumor suppressor gene retinoblastoma (Rb) allowing E2F to dissociate. Once freed, E2F translocates to the nucleus and drives S-phase protein expression which promotes the transition from G1 to S in the cell cycle [32]. miR-30e* inhibition reduced the levels of phosphorylated Rb, but had no effect on total Rb expression levels (Figure 5B; *P ≤0.05). These findings suggest that miR-30e* positively regulates prostate tumor cell proliferation via the NF-κB target gene cyclin-D1. To test this model *in vivo*, miR-30e* resistant IκBα TRAMP C2H tumors as well as corresponding control tumors were immunohistochemically stained for Ki-67 and Cyclin D1 (Figure 5C & 5D). miR-30e* resistant IκBα TRAMP C2H tumors expressed significantly less Ki-67 and cyclin D1 suggesting the miR-30e*: IκBα axis regulates proliferation though the NF-κB target gene cyclin-D1 (Figure 5C & 5D; *** *P* ≤0.005).

In addition to its direct effects on cell proliferation, NF-κB can alter tumor growth and progression through activation of its target genes, including IL-6, VEGF, MMP9 and TNF- α [4, 16, 17]. To test whether miR30e* could be altering the expression of other NF- κB target genes in prostate cancer cells miR-30e* was inhibited and expression was assessed after 24 hours via qRT-PCR. miR-30e* inhibition led to a significant decrease in the expression of TNF- α and VEGF mRNA as well as marginally reduced the expression of IL-10, IL-6 and iNOS (Supplementary Figure 5). MMP9 is another NF-κB target gene that functions to degrade extracellular matrix [27]. MMP9 has been demonstrated to enhance tumor cell invasion, angiogenesis and cell proliferation. Jiang *et al* [27] has shown that miR-30e* augments human glioma tumor growth by NF-κB dependent regulation of MMP9 [27]. miR-30e* inhibition in TRAMP C2H cells did not significantly alter the expression of functional MMP9 (Supplementary Figure 6).

### miR-30e* inhibition sensitizes prostate cancer cells to docetaxel

Docetaxel is the standard of care chemotherapeutic treatment for CaP and has been shown to improve life expectancy [34], yet it's efficacy as a monotherapy is blunted by its high toxicity [35]. Constitutive NF-κB activity contributes to docetaxel resistance in CaP [15]. Treatment of TRAMP C2H cells with docetaxel effectively decreased cell viability while combination treatment of docetaxel and miR-30e* inhibition significantly enhanced docetaxel efficacy (Figure 5E; *P ≤ 0.05), suggesting that inhibition of miR-30e* increases CaP cell sensitivity to chemotherapeutics.

## DISCUSSION

**Figure 6:**
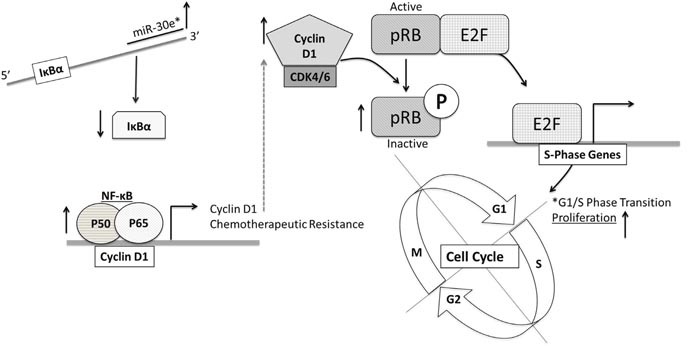
The microRNA-30e*: NF-κB axis regulates prostate cancer cell proliferation and therapeutic resistance miR-30e* targets IκBα mRNA thus increasing NF-κB activation. NF-κB drives chemotherapeutic resistance and also increases cyclin D1 production. Cyclin D1:CDK4/6 phosphorylates pRb allowing it to dissociate from E2F. Free E2F pushes CaP cells from G1 to S phase and drives proliferation and therapeutic resistance.

The goal of this study was to investigate whether miR-30e* contributed to constitutive NF-κB activation in CaP. We report that miR-30e*, a NF-κB activating miR, is hyper-expressed in two murine models of autochthonous CaP relative to healthy controls. We further demonstrated that miR-30e* mediated hyper-activation of NF-κB contributes to CaP cell proliferation and viability. Our results indicated that the effects of miR-30e* in CaP cells are a result of miR-30e* inhibition of IκBα expression. The model we propose (Figure 6) suggests that, miR-30e* becomes hyper expressed in CaP cells as the disease progresses. miR-30e* targets IκBα, thus increasing the level of free NF-κB to translocate to the nucleus. NF-κB drives the expression of cyclin D1, which in combination with CDK4/6, inactivates Rb enhancing CaP cells proliferation and decreases sensitivity to therapy.

Several studies support a role for miR-30e* in NF-κB driven cell proliferation and CaP. Lee *et al*. [35] demonstrated that a decrease in miR-30e* expression correlated with a decrease in proliferation and viability of normal dermal papilla cells. Mortensen *et al*. [36] analyzed the miR profile in CaP patients prior to treatment. miR-30e* was significantly elevated in patients that eventually developed biochemical recurrence following treatment relative to patients who did not. A recent study [37] assessed the molecular profile of 333 primary prostate carcinomas as well as 27 adjacent normal tissues. The results suggest that the majority of primary prostate cancers fall under 7 subtypes which are defined by specific gene mutations/fusions. Analysis of a comprehensive list of microRNA in relation to these specific gene mutations revealed that miR-30e* was overexpressed relative to adjacent healthy tissue in all subtypes and was further elevated in 3/6 of the subtypes defined by having ERG, ETV1 and SPOP fusion proteins respectively. These studies support our finding that miR-30e* is overexpressed in prostate cancer.

Our work suggests that miR-30e* drives CaP progression directly via augmentation NF-κB dependent tumor cell proliferation. In contrast, Jiang *et al*. [27] has shown that miR-30e* contributes to glioma growth and progression indirectly via an increase in NF-κB mediated expression of MMP9, which augments tumor angiogenesis. Inhibition of miR-30e* in CaP cells did not lead to changes in MMP9, suggesting that the effects of miR-30e*/NF-κB are cell-type specific and may be the result of different microenvironments or co-expression of different miRs.

The mechanisms that drive miR-30e* upregulation in CaP are unknown. miR-30e* gene expression has been ascribed to being driven by two different promoters. Patel *et al*. [38] suggests that miR30e is an intronic miR located within the *nuclear factor ϒ C* (*NF-ϒC*) gene however, it is unclear whether the *NF-ϒC* gene promoter or one of several cryptic promoters and possible transcription factor binding sites located within the *NF-ϒC* gene drive miR30e transcription. A separate study by Liao *et al*. [39] reports that miR-30e is driven off its own promoter and can be activated by β-catenin / TCF4. There is also evidence suggesting that the tumor suppressor gene *p53*, a gene often mutated or lost in CaP [40], negatively regulates miR-30e* expression. Hosako *et al* investigated miR profiles from embryos deficient for *p53^−/−^* and discovered increased expression of miR-30e* [41].

While our data demonstrates that NF-κB activation by miR-30e* directly regulates CaP cell proliferation, we also provide evidence that miR-30e* regulates other pathways important in CaP growth. TNF- α and VEGF both have been reported to contribute to prostate cancer. Serum TNF- α is a prognostic factor of disease burden and increases sensitivity to androgen post androgen ablation [17]. VEGF expression in prostate cancer correlates with clinical stage, Gleason score, tumor stage, progression, metastasis, and survival [42]. Traditionally, miRs alter mRNA networks and it would be naïve to think that miR-30e* is an exception. According to microRNA.org, miR-30e* has 7,931 predicted targets and a number of these predicted targets are known to play an important role in CaP progression. The tumor suppressor genes PTEN (Phosphatase and tensin homolog), JNK (c-Jun N-terminal kinase), RB1 as well as an additional inhibitor of NF-κB, NKIRAS1 (NF-κB inhibitor-interacting Ras-like protein 1) are among the putative miR-30e* targets. Wang *et al*. has demonstrated that a conditional deletion of PTEN in the prostate epithelium is sufficient to drive transformation and cancer progression [43]; while Hübner *et al*. indicate that JNK deletion in combination with PTEN deletion develop androgen-independent metastatic CaP more rapidly than the PTEN deletion alone [44]. Rb1 is a vital tumor suppressor that is often lost in advanced CaP. The loss of Rb contributes to tumor progression and androgen receptor activity in CaP [45] yet in inhibition of miR-30e* did not affect total Rb levels (Figure 5B). The importance of the loss of these genes as a function CaP progression in correlation with the increase in miR-30e* throughout disease progression makes these predicted miR-30e* targets very interesting. NKIRAS1 is a potent inhibitor of NF-κB, although the role NKIRAS1 plays in CaP is not well elucidated; the regulation of NF-κB activation by miR-30e* makes this interesting future direction.

miR-30e* is not alone it its ability to regulate NF- κB activation. NF- κB expression is directly affected by miR-9, which inhibits NFKB1 expression [11] and thus NF-κB activity. miR-9 is overexpressed in high grade CaP [46] thus is unlikely to contribute to an increase in NF-κB activation. Interestingly, miR-146 inhibits canonical NF-κB activation [47, 36] and is down regulated in CaP [36], suggesting that this pathways may also contribute to NF- κB activation in CaP.

Docetaxel is an FDA approved therapy for hormone refractory prostate cancer and has been shown to improve life expectancy [34], yet it's efficacy as a monotherapy is blunted by high toxicity [47]. Docetaxel disrupts cell division by preventing depolymerization of microtubules [48]. Work by Domingo-Domenech *et al*. [13] has demonstrated that treatment of castration resistant CaP with docetaxel induces NF-κB activation, which contributes to resistance. Multiple CaP clinical trials have been completed treating patients with bortezomib a non-specific NF-κB inhibitor alone [49] or in combination with the chemotherapeutic docetaxel [50]. These trials have demonstrated that bortezomib alone was capable of slowing the rise of patient's prostate specific antigen (PSA) levels and the combination therapy was able to reduce tumor burden; although there is accompanying treatment toxicity [50]. Our studies indicate that inhibition of miR-30e* effectively lowers the minimal effective dose of docetaxel required to kill CaP cells (Figure 5E). Clinical trials using the RNAi and anti-miRs have demonstrated that durable inhibition is able to be achieved through chemical modification of the oligos. Thus, miR-30e* inhibition has the potential to provide a novel way to target NF-κB hyper-activation in the clinic and provide a window for minimally toxic therapeutic intervention with docetaxel in CaP patients.

## MATERIALS AND METHODS

### Animals and tumor system

C57BL/6J mice were purchased from The Jackson Laboratory (Bar Harbor, ME) and Taconic Laboratory (Hudson, NY). TRansgenic Adenocarcinoma of Mouse Prostate (TRAMP) and SCID mice were bred and maintained at Roswell Park Cancer Institute (RPCI). Hi-MYC and corresponding FVB control mice were purchased from NCI (Frederick, MD) by Dr. Leigh Ellis (RPCI) and were processed and generously given to this study as a gift. All mice were male and housed in microisolator cages in a laminar flow unit under ambient light at 24°C. The RPCI Institutional Animal Care and Use Committee (IACUC) approved all procedures and experiments for this study.

### Reagents and antibodies

Insulin, dihydrotestosterone (DHT), LPS, cell lysis buffer, protease inhibitor cocktail and MTT were purchased from Sigma-Aldrich (St. Louis, MO). NF-κB p65 and Rb specific antibodies as well as the β-Galactosidase staining kit were purchased from Cell Signal (Beverly, MA). Ki-67 antibodies were purchase from Leica Biosystems (Buffalo Grove, IL). BAY 11-7085, phosphatase inhibitor cocktails A and B as well as antibodies specific for cyclin D-1, p-Rb and α-tubulin were purchased from Santa Cruz Biotechnology (Dallas, Texas). Protein assay dye reagent concentrate used for Bradford assays as well as HRP for western blot color development was purchased from Bio-Rad (Hercules, California). DMEM, RPMI640, OPTI-MEM medias, Trypsin-EDTA (0.05%), miR-30e* inhibitor oligos, miR-30e* specific qRTPCR primers, Pre-miR™ miRNA Precursor Molecules—Negative Control #2, lipofectamine 2000, NuPAGE® Novex® 10% Bis-Tris Protein Gels, Nitrocellulose Pre-Cut Blotting Membranes, TRIzol, mirVana™ miRNA Isolation Kit as well as the mirVana™ qRT-PCR miRNA Detection Kit were all purchased from Thermo Fisher Scientific (Waltham, MA). Fetal Bovine Serum (FBS) was purchased from Atlanta Biologicals Inc. (Flowery Branch, GA). Tetracycline-free FBS and the pTetOne vector construct system were both purchased from Clonetech (Mountain View, CA). Luciferase reporter assays and passive lysis buffer were both purchased from Promega (Madison, WI). L-glutamine, penicillin-streptomycin and the selection agent hygromycin B were all purchased from Cellgro (Manassas, VA). Cell lysis buffer #6 and the cleaved-caspase-3 ELISA kit were both purchased from R & D Systems (Minneapolis, MN).

### Cell lines and growth conditions

The murine CaP cell line TRAMP C2H was a generous gift from Dr. Barbara Foster. TRAMP C2H cells were grown in TRAMP media (DMEM supplemented with 10% FBS, 2.5 mg/500mL insulin, 10^−8^ M DHT, L-glutamine and penicillin-streptomycin) in tissue culture flasks at 37°C at 10% CO^2^ [51]. The human CaP cell line PC-3M was purchased from American Type Culture Collection (ATCC; Manassas, VA) and cultured in RPMI640 supplemented with 10% FBS as well as L-glutamine and penicillin-streptomycin. TRAMP C2H cells stably expressing pTetOne-NHA-miR-30e* sensitive-IκBα (WT IκBα) or pTetOne-NHA-miR-30e* resistant IκBα (miR-30e* resistant IκBα) were grown in complete TRAMP media with 10% tetracycline-free FBS in tissue culture flasks in the presence of hygromycin B (3μg/mL).

All cells were maintained and plated for experimentation by harvesting from tissue culture flasks using trypsin (2mL/75cm^2^) and washed with sterile PBS; counted using a hemocytometer and then reseeded in tissue culture flasks or plates with fresh media.

Cells were transfected with miR-30e* inhibitor oligos, scramble oligos, IκBα super repressor, HA-tag wild type IκBα pTetOne or HA-tag miR-30e* resistant IκBα pTetOne using lipofectamine 2000 and OPTI-MEM according to the manufacturer's protocol.

### RNA isolation

Ventral, dorsal and lateral prostates were harvested from 6-, 8-, 12- and 29-week old TRAMP mice and control C57BL/6J mice. Following resection, prostate samples were washed clean with PBS, weighed and flash frozen in eppendorf tubes. Prostates were then transferred to RNAse and DNAse free bags on dry ice and mechanically digested until prostates were a fine powder. The samples were then re-suspended in 10 volumes/tissue mass of cell lysis binding buffer. miRVana RNA isolation protocol provided by the manufacturer was used to harvest RNA. Ventral, dorsal and lateral prostates were harvested from 1-, 3-, 5-, 7- and 9-month old Hi-MYC and FVB controls using Mouse Tumor Model Resource (RPCI). Prostates were homogenized in 1 mL TRIzol and protocol provided by manufacturer was used to harvest RNA.

### Quantitative real-time PCR (qRT-PCR)

qRT-PCR was performed using miRVana qRT-PCR miRNA Detection kit (Figure 1A) as well as iScript Select cDNA synthesis kit followed by SSO advanced SYBR qRTPCR reagents (Figure 1B) using primers specific for miR-30e* and U6 (Thermo Fisher Scientific, Waltham, MA). Raw data was analyzed using the 2-^ΔCq^ formula.

### MTT assays

TRAMP C2H cells were plated at concentration of 5 × 10^4^ cells / well for day 1 and 2.5 × 10^4^ cells / well for day 2 in duplicate for technical replicates and were plated at a concentration of 1 × 10^5^ cells/well in 6 well plates. PC-3M cells were plated at a concentration of 2 × 10^5^ cells / well for day 1 and 1 × 10^5^ cells / well for day 2 in duplicate for technical replicate and were plated at a concentration of 2 × 10^5^ cells/well in 6 well plates for Figure 3D. Cells were allowed to adhere overnight, washed with PBS and then 2 mL of complete media was added back to each well. Cells were then transfected with either a miR-30e* inhibitor or control scramble oligos or treated with Bay 11-7085 inhibitor. Twenty-four or 48 hours later 400 μL of 5 mg/mL 3-(4, 5-dimethylthiazol-2-yl)-2, 5-diphenyltetrazolium bromide (MTT) was added to each well and left to develop in the dark at 37°C for 4 hours. Following the 4 hour development period formalin crystals in media were pelleted and suspended in 1 mL of acidified isopropanol, this was added to each well to dissolve crystals that were adherent to the plate. This was left to sit for 30 minutes shaking and then samples were mixed up and down via pipetting until thoroughly suspended. Two hundred μL from each sample were transferred to individual wells in a 96 well plate where absorbance at 570 nM was recorded on a spectrophototer. Experimental values were normalized to scramble oligo treated controls.

### Senescence associated β- gal staining

TRAMP C2H cells were plated at a concentration of 5 × 10^4^ cells / well in 6 well tissue culture plates. Cells were left to adhere overnight, rinsed with sterile PBS and then fresh media was added to each well. Cells were then transfected with miR-30e* inhibitor. Twenty-four hours later cells were rinsed with sterile PBS twice, fixed and treated with β-galatosidase staining solution overnight at 37°C. Each condition was plated in duplicate and counted in triplicate under light microscopy 200x.

### Cleaved-caspase 3 ELISA

TRAMP C2H cells were plated at 5 x10^5^ cells/10 cm tissue culture dish in 10 mL media. Cells were left to adhere overnight, rinsed with PBS and then fresh media was added. Cells were then transfected with miR-30e* inhibitor. Twenty-four hours later cells were rinsed with PBS twice and harvested. Protein lysates were generated using cell lysis buffer #6 (R&D Systems Minneapolis, MN). The ELISA was purchased from R&D Systems and run according to the provided protocol. One hundred and twenty-five μg of protein was used per well and all samples were run in duplicate.

### *In vitro* Tramp C2H Ki67 staining

TRAMP C2H cells were plated at a concentration of 1×10^6^ cells / 10 cm tissue culture dish in 10 mL media. Cells were left to adhere overnight, rinsed with PBS and then fresh media was added to each well. Cells were then transfected with miR-30e* inhibitor and 24 hours later cells were rinsed with sterile PBS twice and harvested. Cells were then pelleted, fixed in ice-cold ethanol, re-pelleted and then overlayed with agar. Tissues were processed and embedded in paraffin and then sectioned at 5 microns. Slides were de-parafinized in several baths of xylene and then rehydrated in graded alcohols followed by ddH2O. Slides were incubated in 1x pH6 citrate buffer (Invitrogen, Grand Island, NY) for 20 minutes. Slides were washed, blocked and then stained with Ki67 antibody. For signal enhancement, ABC reagent (Vector Labs, Burlingame, CA) was applied for 30 minutes. To reveal endogenous peroxidase activity, slides were incubated with DAB substrate (Dako, Carpinteria, CA) for 5 minutes and then counterstained with DAKO Hematoxylin for 20 seconds. Slides were dehydrated through several baths of graded alcohols and xylenes.

### NF-κB activity assay

TRAMP C2H cells were plated in 6 well tissue culture plates at a concentration of 5 × 10^4^ cells/well. Cells were allowed to attach overnight and then transfected with 2.5 μg of a NF-κB reporter luciferase construct. Six hours later cells were washed and then transfected with miR-30e* inhibitor for 24 hours. Cells were then washed in PBS and harvested in 500 μL 1x passive lysis buffer. Luciferase was quantified using promega luciferase assay kit on a luminometer. Experimental values were recorded relative to untreated control samples.

### Trypan blue exclusion assay

TRAMP C2H and PC3M cells were plated at a concentration of 2.5 × 10^4^ cells / well and 2 × 10^5^ cells / well respectively in 6 well plates and left to adhere overnight. Cells were washed with sterile PBS and 2 mLs of fresh media was added back to each well. Cells were then transfected with 2.5 μg of IκBα super repressor construct using lipofectamine 2000 according to the manufacturers’ protocol. Twenty-four hours later cells were harvested using Trypsin-EDTA (0.05%) and counted via trypan blue exclusion on hemocytometers.

### Western blot

To generate protein lysates cells were plated at 5 × 10^5^ and left to adhere overnight on 10 cm dishes. Cells were harvested and incubated with cell lysis buffer supplemented with a protease inhibitor cocktail and phosphatase inhibitor cocktails A and B on ice for 30 min. Lysates were pelleted at 14,000 rpm/20 min and the aqueous layer was harvested. The amount of protein was quantified using Bradford assays. Twenty μg of protein was run per sample on 10% bis-tris gels and then transferred to nitrocellulose membranes. Blots were probed with cyclin D-1, p-Rb, HA-tag, NF-κB p65, or α-tubulin specific antibodies. HRP development was recorded using a Bio-Rad imager and quantified using Quantity One software (Bio-Rad, Hercules, California). All values were normalized to α-tubulin; experimental values were then normalized to control values.

### Tumor inoculation and doxycycline treatment

miR-30e* resistant Tramp C2H cells were harvested from tissue culture flasks *in vitro* using Trypsin-EDTA (0.05%) during log phase growth. Cells were washed 3x in sterile room temperature PBS and then resuspended at a concentration of 1×10^6^ / 100μL in sterile PBS. Tumor cells were inoculated in 100μL subcutaneously in the flanks of C57BL/6 mice using 18G needles due to the large size of the cell line. Tumors were monitored via hand calipers and allowed to establish to 100mm^3^ to control for tumor establishment variability and then experimental mice were fed doxycycline chow (200 mg/kg). Experimental endpoint was when the tumor volumes reached 800mm^3^ in accordance with IACUC protocol.

### Tumor NF-κB p65, Ki67 and cyclin D1 immunohistochemistry

miR-30e* resistant Tramp C2H tumors were resected when tumor volume reached 800mm^3^ from euthanized mice. Tumors were fixed overnight in 10% buffered formalin. Formalin-fixed paraffin sections were cut at 4μm, placed on charged slides, and dried at 60°C for one hour. Slides were cooled to room temperature, deparaffinized in three changes of xylene, and rehydrated using graded alcohols. For antigen retrieval, slides were heated in a steamer for 30 minutes in citrate buffer pH=6 (BioCare Medical; catalog #CB910) and allowed to cool for 20 minutes. Endogenous peroxidase was quenched with aqueous 0.3% H2O2 for 10 minutes and washed with PBS/T. Slides were loaded on a Dako autostainer and serum free protein block (Dako; catalog #X0909) was applied for 5 minutes, blown off, and the Ki67 antibody (Thermo Scientific; catalog# RM-9106-S1) was applied at 1/150 (1 μg/ml Rb IgG) or Cyclin D1 antibody (Abcam; catalog #ab16663) applied at 1/100 (1 μg/ml Rb IgG) for one hour or NF-κB p65 antibody (Abcam; catalog #ab7970) was applied at 1/4000 (0.05 ug/mL Rb IgG) over night at 4 degrees C. Biotinylated goat anti-rabbit (Vector Labs; catalog #BA-1000) was then applied for 30 minutes followed by Elite ABC (Vector Labs; catalog# PK6100) for 30 minutes or rabbit envision/labeled polymer HRP anti-rabbit (Dako; catalog #K4003) was then applied for 30 minutes. DAB (Dako; catalog #K3468) applied for 5 minutes, was used for chromogen visualization. Lastly, the slides were counterstained with Hematoxylin, dehydrated, cleared and cover slipped. Slides were scanned and digitalized using Aperio Digital Pathology Slide Scanner (Leica). Quantification of IHC was performed by visually counting positively stained cells in sections from slides that had been digitalized via the Aperio Digital Pathology Slide Scanner (Leica). Cells that displayed positive staining for Ki-67 and cyclin-d1 were quantified while NF-*κ*B p65 staining was only counted when it colocalized with the nuclear staining. Three sections from each tumor were quantified for Ki-67 and cyclin D1 while five sections from each tumor were quantified for NF-*κ*B p65.

## SUPPLEMENTARY MATERIALS FIGURES


