# Supplementary material for: miR-30e* is overexpressed in prostate cancer and promotes NF-κB-mediated proliferation and tumor growth
Source: Oncotarget. 2017 Jun 28;8(40):67626–38. doi: 10.18632/oncotarget.18795 (PMC5620198; doi:10.18632/oncotarget.18795)
Supplement: Supplementary file 1 [file oncotarget-08-67626-s001.pdf]

miR-30e* is overexpressed in prostate cancer and promotes NF-κB-mediated proliferation and tumor growth

SUPPLEMENTARY MATERIALS

Reagents and antibodies

Antibody specific for HA-tag, α-Tubulin and IκBα as well as the NF-κB inhibitor Bay-11-7085 were purchased from Santa Cruz Biotechnology (Dallas, Texas). An additional IκBα antibody as well as the p-IκBα and NF-κB p65 antibodies were purchased from Cell Signaling Technology (Danvers, MA). iScript Select cDNA synthesis kit, iScript cDNA synthesis kit, SSOadvanced universal SYBR green supermix, Protein assay dye reagent concentrate used for Bradford assays as well as HRP for western blot color development was purchased from Bio-Rad (Hercules, California). Trizol, miRVana microRNA isolation kit, NuPAGE® Novex® 10% Bis-Tris Protein Gels, Nitrocellulose Pre-Cut Blotting Membranes, Novex 10% Zymogram (Gelatin) Gels, Novex® Zymogram Developing Buffer (10X), Novex® Zymogram Renaturing Buffer (10X), TOPO® TA Cloning® Kit, Platinum® Taq DNA Polymerase and SimplyBlue™ SafeStain were all purchased from Thermo Fisher Scientific (Waltham, MA). Tet-One™ Inducible Expression System was purchased from Clontech Laboratories (Mountain View, CA). pSELECT HA-Tag vector was purchased from InvivoGen (San Diego, CA). β-actin specific antibodies as well as Lipopolysaccharides (LPS) were purchased from Sigma-Aldrich (St. Louis, MO). Primers were purchased from Integrated DNA Technologies (Coralville, IA). Luciferin and stop and glow renilla luciferase were purchased from Gold Bio technologies and Promega (Madison, WI) respectively.

Quantitative real-time PCR (qRT-PCR)

RNA was harvested using trizol and miRVana microRNA isolation kits. RNA was quantified using a Smart Spec Plus (BioRad). cDNA synthesis was performed using 50 ng total RNA per reaction utilizing iScript Select cDNA synthesis reagents as well iScript cDNA synthesis reagents. Two uLof the cDNA synthesis reaction was used per qRT-PCR reaction using SSOadvanced SYBR green supermix. qRT-PCR was also performed using miRVana qRT-PCR miRNA Detection kit using primers specific for miR-30e*and U6 (Thermo Fisher Scientific, Waltham, MA). Cq values were analyzed using the $2^{-\Delta Cq}$ formula.

qRT-PCR primers

TNF-α

Forward: CCA CGC TCT TCT GTC TAC TG
Reverse: GGA ACT TCT CAT CCC TTT GG

iNOS

Forward: ACA AGC TGC ATG TGA CAT CG
Reverse: GGC AAA GAT GAG CTC ATC CA

IL-10

Forward: GAG ACT TGC TCT TGC ACT ACC
Reverse: CTC TCT TTT CTG CAA GGC TG

TGFβ

Forward: CGT CAG ACA TTC GGG AAG A
Reverse: CGT ATC AGT GGG GGT CAG CA

IL-6

Forward: GAC AAA GCC AGA GTC CTT CAG AGA G
Reverse: CTA GGT TTG CCG AGT AGA TCT C

VEGF

Forward: GAG GAT GTC CTC ACT CGG ATG
Reverse: GTC GTG TTT CTG GAA GTG AGC AA

Western blot

For experiments investigating effectiveness of Bay 11-7085, TRAMP C2H and PC3M cells were plated at a concentration of 2 x 10^5 and 3 x 10^5 cells/well in 6 well plates respectively. Cells were pre-incubated with either 10µM, 50µM Bay 11-7085 or equal volume ethanol carrier as a control prior to LPS (1µg/mL) stimulation. Samples were harvested 30 minutes following LPS stimulation. Cells were harvested and incubated with cell lysis buffer supplemented with a protease inhibitor cocktail and phosphatase inhibitor cocktails A and B on ice for 30 min. To generate protein lysates from tumors; tumors were excised and washed with PBS on ice. Tumors

were mechanically digested and then incubated with cell lysis buffer supplemented with a protease inhibitor cocktail and phosphatase inhibitor cocktails A and B on ice for 30 min. Lysates were pelleted at 14,000 rpm/20 min and the aqueous layer was harvested. The amount of protein was quantified using Bradford assays. Twenty µg of protein was run per sample on 10% bis-tris gels and then transferred to nitrocellulose membranes. Blots were probed with HA-tag, IκBα, p-IκBα, α-Tubulin, NF-κB p65 or β-actin specific antibodies. HRP development was recorded using a Bio-Rad imager and quantified using Quantity One software (Biorad- Hercules, California). α-Tubulin and β-actin were run as housekeeping controls.

N-HA pTetOne constructs

The IκBα gene and 3'UTR was isolated from murine genomic DNA by PCR using primers forward- 5'-CAT GTT TCA GCC AGC TGG GC-3' and reverse- 5'-GGC GAC ACA GAC CTT AGT TGG-3' and inserted into a TOPO 2.1 plasmid. After sequencing, the start site was removed and the RE sites BamH1 and NheI were added to the 5' and 3' ends of IκBα respectively via PCR using the primers forward- 5'- GGATCCTTTCAGCCAGCTGG-3' and reverse- 5'-GCTAGCGGCTCTTACATGGGA-3'. The fusion gene encoding N-HA IκBα was inserted into the pTetOne plasmid to generate a doxycycline inducible N-HA IκBα. The miR-30e* target sequence located on the 3'UTR was mutated using site directed mutagenesis and is referred to as N-HA miR-30e* resistant IκBα.

Co-immunoprecipitation assays

TRAMP C2H expressing miR-30e* resistant and sensitive IκBα were plated at a concentration of 5 x 10^5 cells / 10 cm plate. Cells were left to adhere overnight, washed with sterile PBS and then fresh media was added to each well. Doxycycline hyclate was added to the desired plates at a concentration of 100 ng/mL and left to incubate for 24 hours. Cells were then gathered and lysates were harvested using Cell lytic MT Cell lysis Reagent (Sigma-Aldrich, St. Louis, MO) supplemented with Protease Inhibitor Cocktail, Phosphatase Inhibitor Cocktail A and Phosphatase Inhibitor Cocktail B. Protein concentrations were determined using Bradford assays and 250µg protein was pre-cleared of Ig with 50µL Protein G-Agarose slurry (Roche, Branford, CT) by incubating at 4°C for 2 hours. Slurry was pelleted and cleared lysate was extracted. Three µg of HA-tag antibody was added to lysates and incubated with shaking at 4°C. 50µL Protein G-Agarose slurry was incubated on shaker at 4°C for 2 hours. Slurry was pelleted and then eluted in cell lysis buffer by boiling at 95°C for 5 minutes. Samples were then run using standard western blot protocols.

Zymography

To generate cell supernatants, cells were plated at 1 x 10^6 and left to adhere overnight on 10 cm dishes. Cells were subsequently treated with miR-30e* inhibitor and then supernatants were harvested 24 hours later. Supernatants were spun down to remove cellular contamination. Samples were loaded onto 10% Zymogram (Gelatin) gels and run at 125 Voltz / 95 minutes. Gels were incubated with 1x renaturing buffer while shaking for 30 minutes, subsequently the gels were washed and incubated in 1x developing buffer in a 37°C incubator for 48 hours. Gel was stained with simply blue stain for 60 minutes then scanned and analyzed with ImageJ software.

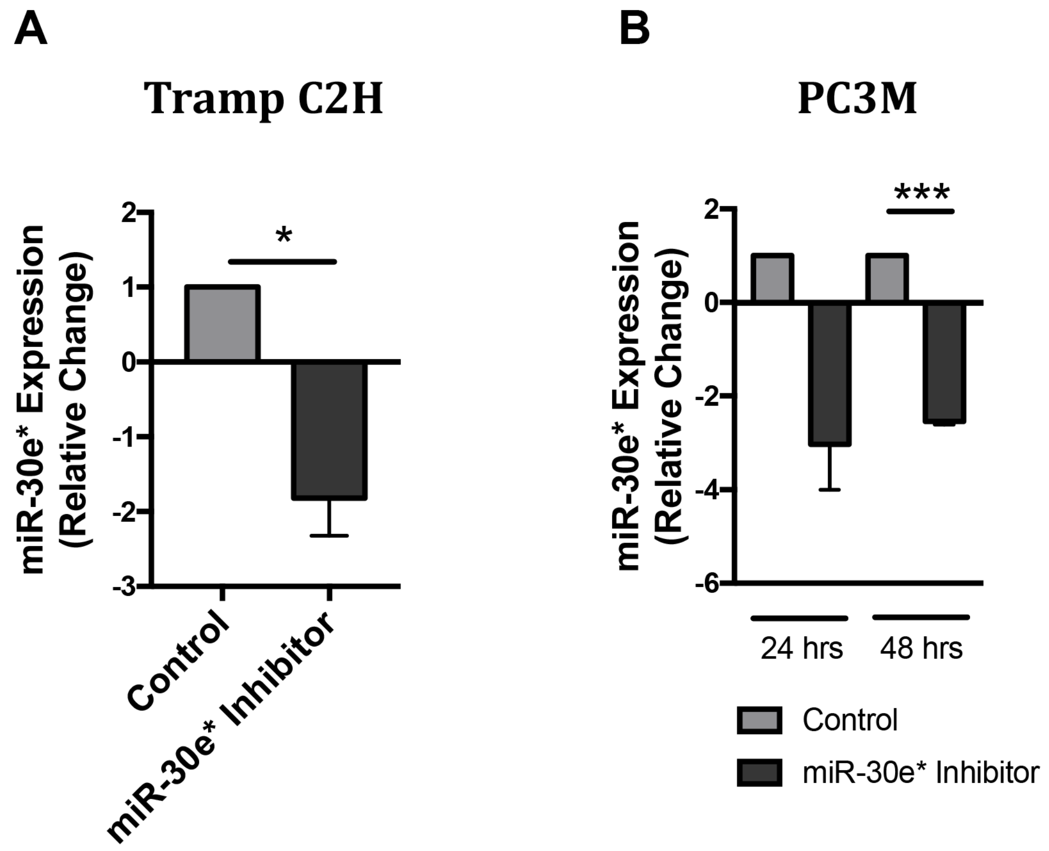

Supplementary Figure 1: Confirmation that mIR-30e* inhibitor successfully inhibits miR-30e*. Tramp C2H **(A)** and PC3M **(B)** cells were transfected with miR-30e* inhibitor and miR-30e* and U6 housekeeping control were assessed 24 (A & B) and 48 (B) hours later. Samples were harvested from 6 well plates in cell lysis binding buffer. RNA was harvested using miRVana microRNA isolation kit. cDNA specific to miR30e* and U6 were generated using iScript select reagents and subsequent qRTPCR was performed using SSO advanced reagents. Raw data was analyzed using the 2^{-dCq} formula and reported as relative to untreated control. Student t-tests were performed, Error bars represent SEM, n = 2, *$P \leq 0.05$ ***$P \leq 0.001$.

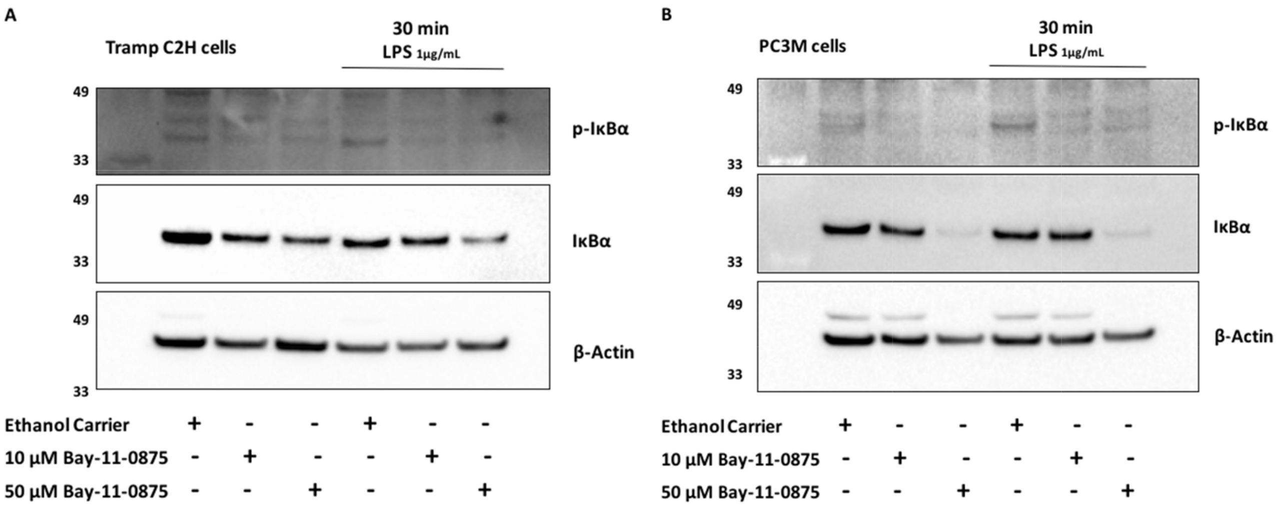

Supplementary Figure 2: NF-κB inhibition via Bay 11-7085 was confirmed in both TRAMP C2H and PC3M cells. Bay 11-7085 acts to inhibit NF-κB by acting as an irreversible inhibitor of IκBα phosphorylation. LPS is a classical NF-κB activator that acts by rapidly (30 min) inducing the phosphorylation and subsequent degradation of IκBα. TRAMP C2H **(A)** and PC3M **(B)** cells were pre-incubated with either 10µM, 50µM Bay 11-7085 or equal volume ethanol carrier as a control prior to LPS (1µg/mL) stimulation. Samples were harvested 30 minutes following LPS stimulation. Western blot analysis was used to assess p-IκBα, IκBα and β-actin was used as a housekeeping loading control. Representative blot depicted of an experiment that was repeated 3 times in both TRAMP C2H and PC3M.

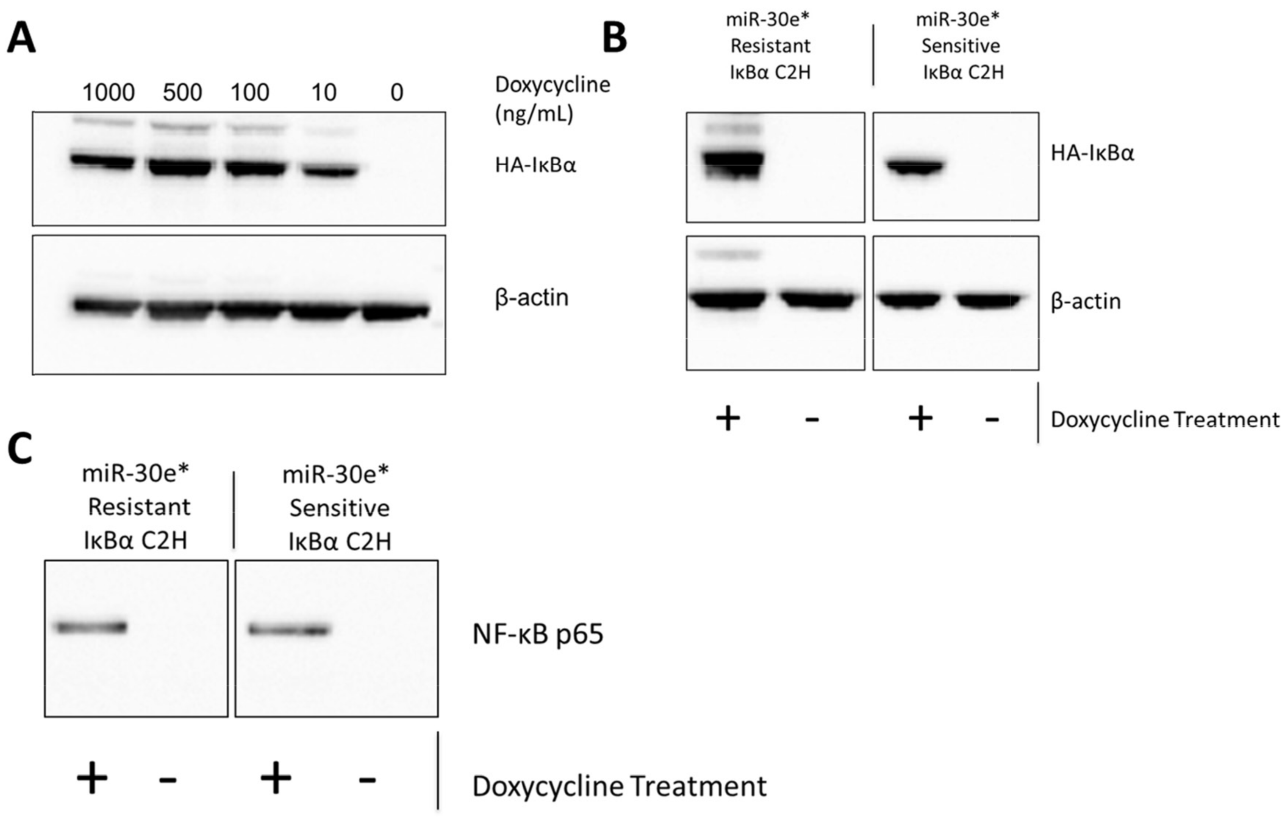

Supplementary Figure 3: HA- IκBα induction and function following doxycycline hyclate treatment of N-HA miR-30e* sensitive and resistant IκBα transfected C2H cells. **(A)** HA- IκBα induction was analyzed in miR-30e* sensitive IκBα transfected C2H cells via western blot analysis following 24-hour stimulation of increasing doses of doxycline hyclate. β-actin was also analyzed for loading control. **(B)** HA-IκBα induction in N-HA miR-30e* sensitive and resistant IκBα C2H clones. HA- IκBα induction was analyzed in miR-30e* sensitive and resistant IκBα transfected C2H cells via western blot analysis. Cells were stimulated with 100 ng/mL sterile doxcycine hyclate for 24-hours. β-actin was also analyzed for loading control. **(C)** N-HA miR-30e* sensitive and resistant IκBα transfected C2H cells were stimulated with 100 ng/mL sterile doxcycine hyclate for 24-hours. HA-tag antibody was used for co-immunoprecipitation capture. NF-κB p65 was analyzed from co-immunoprecipations via western blot analysis.

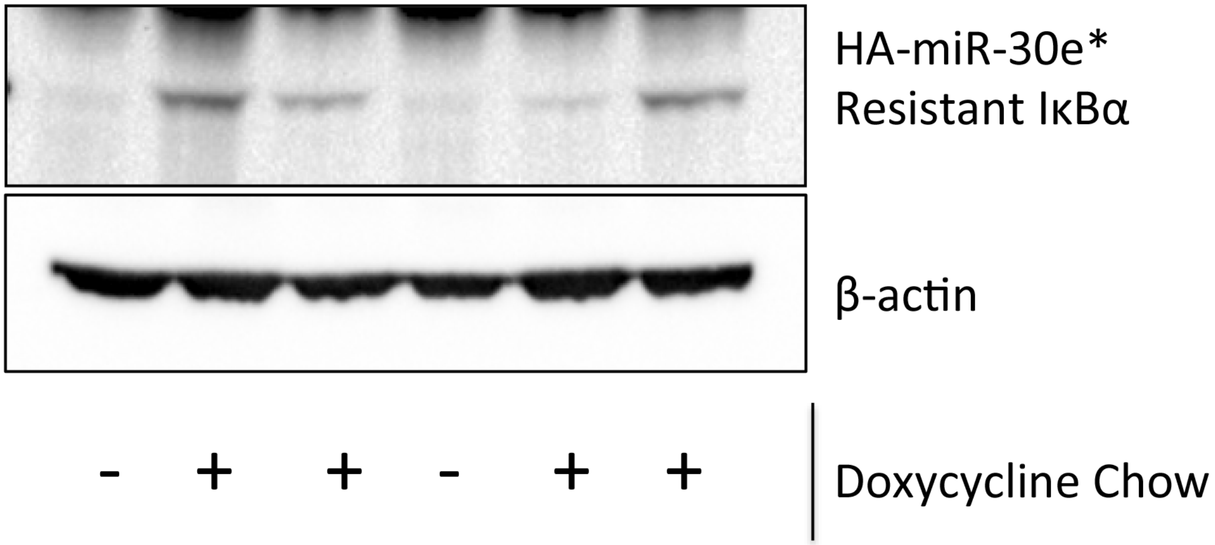

Supplementary Figure 4: HA-IκBα induction in N-HA miR-30e* miR-30e* resistant IκBα C2H tumors following administration doxycycline chow. HA-IκBα induction was analyzed in miR-30e* resistant IκBα transfected C2H cells via western blot analysis. Tumors were grown until 100 mm^3 after which mice were fed doxycycline chow or corresponding normal chow. The endpoint for the experiment was when tumors reached 600-800 mm^3, subsequently tumors were harvested and lysates were generated. Western blots were probed with HA antibody and β-actin was also used for a loading control.

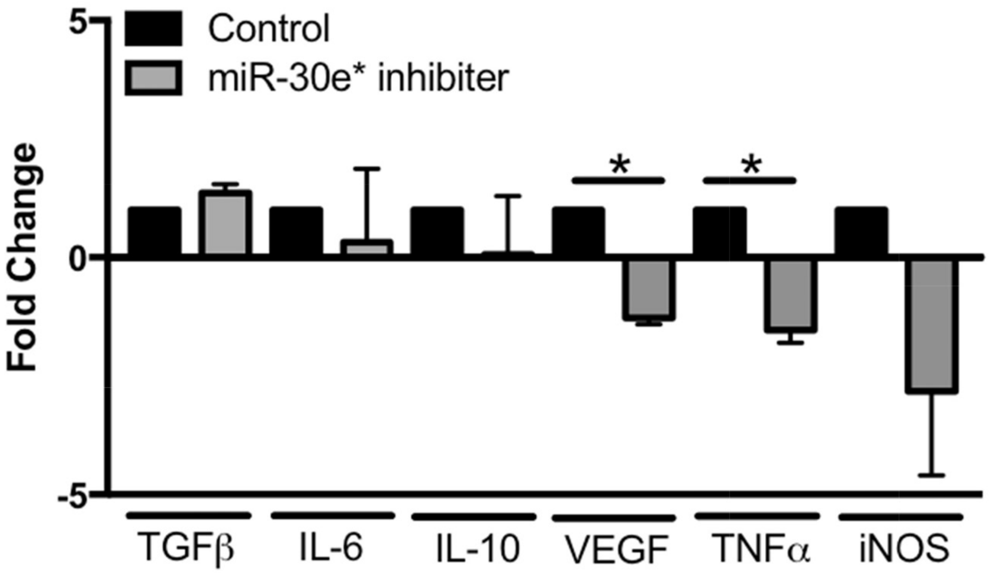

Supplementary Figure 5: NF-κB target genes are downregulated following miR-30e* inhibition. TRAMP C2H cells were plated in 6 well plates at a concentration of 1 x 10^5. Cells were left to adhere overnight and then transfected with miR-30e* inhibitor or scramble oligo control using lipofectamine 2000 reagents for 24 hours. Cells were then rinsed and lysed using lysis binding buffer. RNA was harvested using miRVana microRNA isolation kit, cDNA was generated cDNA synthesis kit and qRTPCR was done using SSO advanced reagents. Raw data was analyzed using the 2^{-dCq} formula and reported as relative to scramble control. Student t-tests were performed, error bars represent SEM, n = 2, *$P \leq 0.05$.

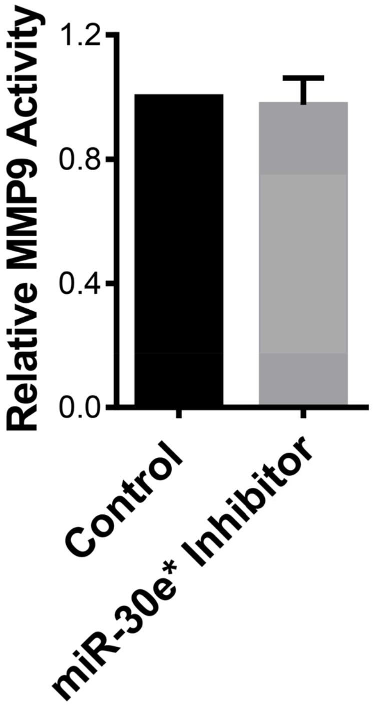

Supplementary Figure 6: MMP9 zymography in control and miR-30e* inhibitor treated C2H cells. C2H cells were treated for 24 hours with miR-30e* inhibitor and MMP9 proteinase activity was assessed via zymography. Results are depicted as MMP9 proteinase activity relative to control C2H cells. Student t-tests were performed, error bars represent SEM, n=4 $P > 0.05$.
